# Candidate gene-based association mapping of growth and wood quality traits in *Eucalyptus globulus* Labill

**DOI:** 10.1186/1753-6561-5-S7-O15

**Published:** 2011-09-13

**Authors:** Saravanan Thavamanikumar, Josquin Tibbits, Luke McManus, Peter Ades, Desmond Stackpole, Sara Hadjigol, René Vaillancourt, Peng Zhu, Gerd Bossinger

**Affiliations:** 1 Department of Forest and Ecosystem Science, The University of Melbourne, Water Street, Creswick, Victoria 3363, Australia and Co-operative Research Centre for Forestry, Private Bag 12, Hobart 7001, Tasmania, Australia; 2 Department of Forest and Ecosystem Science, The University of Melbourne, Water Street, Creswick, Victoria 3363, Australia. Present address: Department of Primary Industries, Biosciences Research Division, Victorian AgriBiosciences Centre, 1 Park Drive, La, Australia; 3 School of Plant Sciences, University of Tasmania, Private Bag 55, Hobart 7001 Tasmania, Australia. Present address: Scion, Te Papa Tipu Innovation Park, 49 Sala Street, Rotorua 3010, Private Bag 3020, Rotorua 3046, New Zealand; 4 School of Plant Sciences, University of Tasmania, Private Bag 55, Hobart 7001 Tasmania, Australia and Co-operative Research Centre for Forestry, Private Bag 12, Hobart 7001, Tasmania, Australia; 5 South China Botanical Garden, Chinese Academy of Sciences, Guangzhou, Guangdong 510650, People Republic of China

## Background

The identification of polymorphisms that underlie complex phenotypic traits presents new and exciting challenges to molecular genetics. Association mapping, which uses linkage disequilibrium (LD) to map trait variation with nucleotide polymorphisms, has proved suited for this purpose in outcrossed tree species. Association mapping can be undertaken either at a candidate gene level or at the whole-genome level however; until sufficiently dense marker assays are developed the candidate gene approach will remain the most effective way of dissecting complex traits in tree species. To date, this approach has led to the identification of several quantitative trait nucleotides (QTN) that associate with a variety of breeding traits including, early wood specific gravity, percentage latewood [1], microfibril angle [2], cellulose [3]and carbon isotope discrimination [4].

## Aim

Our research aims to identify single nucleotide polymorphism (SNP) markers using candidate gene-based association mapping that can predict growth and wood quality in *Eucalyptus globulus*.

## Materials and methods

A *Eucalyptus globulus* provenance-progeny trial, planted in 1989 near Latrobe in north-central Tasmania, Australia, by Gunns Ltd, was used as the association discovery population. An eight-year-old Southern Tree Breeding Association (STBA) breeding trial growing near Frankland, Western Australia, is being used for validating marker-trait associations. Twenty functional candidate genes for wood and fiber formation, were selected for this study. SNPs were discovered by direct sequencing of PCR products from 11 to 28 trees.

The iPLEX Gold assay (Sequenom Inc.)was used to genotype 98 selected polymorphisms in up to 385 individuals from the discovery population. This subset includes individuals from eight races of *E. globulus*. Linkage disequilibrium between SNPs was estimated using GEVALT [5]and Hardy-Weinberg equilibrium was estimated using FSTAT ver 2.9.3.2 [6]respectively.

To account for genetic structure, 18 SSR markers were genotyped in all the discovery samples. Ancestry (Q) co-efficients were estimated using the model-based clustering method as implemented in STRUCTURE [7]. A matrix of pairwise kinship coefficients (K) was calculated as described in Ritland [8], using the software SpaGeDi [9]. Marker-trait associations were tested using a mixed linear model (MLM) [10], which accounted for both population structure and familial relatedness using TASSEL version 2.0.1 [11].

## Results

At a 5% significance level, only 4 SNPs deviated from Hardy Weinberg expectations in more than one race. With all races pooled, LD between SNPs was very low with only 1.8% of the pairwise comparisons having r^2^ values greater than 0.33. Only where r^2^ is greater than 0.33 is there sufficiently strong LD to be useful for association mapping [12]. Only one pair of SNPs, *EgCSA3*_4186 and *EgMYB2*_1380 between genes was found to be in LD (r^2^ > 0.5). When LD was computed within races 1.3 to 2.7% of the pairwise comparison had r^2^ values greater than 0.33.

Of the 98 polymorphisms tested against 12 traits, 33 associated significantly (P < 0.05) with one or more traits giving a total of 62 associations. Individual polymorphisms explained between 0.9 and 3.8% of the phenotypic variation observed. Marker-trait associations found in the discovery population are currently being validated by testing their consistency using the validation population.

## Conclusions

Candidate gene based association mapping studies are a useful means of dissecting complex quantitative traits in species like *E. globulus* with low LD and high nucleotide diversity. This study, like other tree association mapping studies, shows that the percentage of phenotypic variation explained by a single polymorphism will often be small. This is not unexpected because of the complex nature of most wood quality traits. The small proportion of the phenotypic variation explained so far using association mapping should not detract from its future use in tree breeding, since genotyping costs are expected to fall while throughput increases, thereby facilitating larger scale association mapping efforts in the near future.
